# Pyrolysis of Biomass Impregnated With Ammonium Dihydrogen Phosphate for Polygeneration of Phenol and Supercapacitor Electrode Material

**DOI:** 10.3389/fchem.2020.00436

**Published:** 2020-05-19

**Authors:** Kai Li, Bo Wang, Dana Bolatibieke, Dong-hong Nan, Qiang Lu

**Affiliations:** National Engineering Laboratory for Biomass Power Generation Equipment, North China Electric Power University, Beijing, China

**Keywords:** biomass, NH_4_H_2_PO_4_, pyrolysis, phenol, supercapacitor

## Abstract

A new method was proposed for polygeneration of phenol and supercapacitor electrode material from pyrolysis of biomass impregnated with ammonium dihydrogen phosphate (NH_4_H_2_PO_4_). The pyrolysis experiments were executed to demonstrate the product distributions under different NH_4_H_2_PO_4_-to-poplar (PA-to-PL) ratios and pyrolysis temperatures in a lab-scale device. The results revealed that the phenol yield attained its optimal value of 4.57 wt% with a satisfactory selectivity of 20.09% at 500°C under PA-to-PL ratio of 0.6. The pyrolytic solid product obtained at this condition was then subjected to high temperature activation directly without additional activators to prepare N and P co-doped activated carbon (NPAC) as supercapacitor. The physicochemical analysis of NPAC showed that the N and P contents in NPAC reached 3.75 and 3.65 wt%, respectively. The electrochemical experiments executed in a three-electrode system indicated that the NPAC exhibited promising electrochemical performance with a satisfactory capacitance of 181.3 F g^−1^ at 1 A g^−1^.

## Introduction

Lignocellulosic biomass can be quickly converted into liquid, gaseous and solid products by fast pyrolysis technology (Lu et al., 2018b; Wang et al., 2018a). The efficient utilization of the pyrolytic products directly determines the application of this technology. Among the three biomass pyrolytic products, the liquid product (bio-oil) is usually the dominant product with potential applications as liquid fuels or raw chemical materials (Kan et al., 2016; Chen et al., 2019). Particularly, there are various value-added chemicals in bio-oil, which allows their separation and purification to obtain specific compounds (Brueckner et al., 2020). However, the contents of most chemicals in bio-oil are extremely low, making the conventional bio-oil fairly difficult to be utilized (Chen et al., 2014; Muneer et al., 2019). Therefore, tireless and unremitting endeavors have been devoted to regulating the biomass pyrolysis process pertinently for preparing specific bio-oils abundant in target valuable products, such as levoglucosenone (Ye et al., 2017), furfural (Su et al., 2020), phenol (Chang et al., 2018), and so on.

Phenol is one of high value-added compounds derived from biomass pyrolysis, widely used in synthetic fibers, medicine, pesticides, and so on. It is mainly decomposed from lignin (Lazaridis et al., 2018). Because of the irregular lignin structures, fast pyrolysis of lignin or biomass will inevitably form hundreds of phenolic compounds in bio-oil, rather than any specific phenolics (Dong et al., 2012; Duan et al., 2019). Hitherto, various researches have been concentrated on the selective pyrolysis of biomass to produce mixed or specific phenolic compounds (Yaman et al., 2018). Lu et al. (2013) employed K_3_PO_4_ for the biomass pyrolysis to prepare phenolics. The maximum phenolics content of 64.2% (in GC peak area%) was obtained at 500°C with the K_3_PO_4_ content of 50.51 wt%. Among the phenolics, phenol was a major product with a high peak area% of 14.3%. Bu et al. (2012) conducted the biomass pyrolysis experiments catalyzed by acid-washed activated carbon (AC). Mixed phenolics with the concentration of 66.9% (in peak area%) were obtained, accompanied with the phenol concentration of 38.9%.

Although mixed phenolics could be selectively prepared from biomass catalytic pyrolysis and phenol was the main compound in the mixed phenolics, the variety of phenolics mixture in the liquid product would inhibit the further purification of phenol. Therefore, it is fatal to further promote the yield and selectivity of phenol. Currently, only limited researches have been performed on the selective preparation of phenol. Our group put forward a creative method to obtain phenol from catalytic decomposition of biomass by impregnation of K_3_PO_4_ or blended with magnetic K_3_PO_4_/Fe_3_O_4_ solid catalyst under hydrogen atmosphere (Lu et al., 2018c; Zhang et al., 2019). The phenol yields reached 5.3 and 4.3 wt%, respectively, both with excellent selectivity of ~18%. The hydrogen source in the pyrolysis process facilitated the elimination of substitutes of phenolic compounds to generate phenol (Liu et al., 2014; Zhang et al., 2019). Differed from conventional techniques to produce phenol from lignin, Zhang et al. (2018a) established a new and unique system to selectively produce phenol from non-lignin materials, such as cellulose. With the assistant of AC prepared by H_3_PO_4_ activation, the phenol from cellulose pyrolysis achieved an excellent selectivity of 99.02% (in peak area%) under 450°C. Wherein, the P-containing groups in the AC were supposed to be responsible for the generation of phenol.

In addition to the liquid product, solid product (char) is usually an important by-product of biomass fast pyrolysis process. Char is widely used as solid fuels or further processed to ACs (Xu et al., 2020), carbon-based fertilizers (Liang et al., 2016), supercapacitor electrode materials (Lu et al., 2018a), and so on. The utilization of char to prepare supercapacitor electrode materials has been drawing increasing attention due to the availability, low cost and renewability of raw materials (Wang and Wang, 2019; Kim et al., 2020). However, raw char product obtained directly from lignocellulosic biomass pyrolysis process exhibited unsatisfactory capacitive performance because of the limited surface area and active sites (Zhao et al., 2017). Therefore, activation and incorporation of heteroatoms (N, P, S, etc.) were adopted to improve porosity and functional groups of char materials (Wang et al., 2018b). Chen et al. (2016) synthesized the N doped carbon materials by subjecting biomass successively to pyrolysis in the NH_3_ atmosphere and KOH activation. The obtained AC possessed high nitrogen and specific surface area (SSA) with the specific capacitance (C_g_) of 187 F g^−1^ at 1 A g^−1^. Wang et al. (2013a,b) synthetized two N and P co-doped activated carbons (NPACs) for supercapacitors by carbonization of polyaniline impregnated with H_3_PO_4_ and glucose with (NH_4_)_3_PO_4_. The corresponding C_g_s of the NPACs at 0.05 A g^−1^ could reach 154.4 and 183.8 F g^−1^, respectively. All of these studies suggested that incorporating N and P into carbon materials would be conducive to optimizing the electrochemical performance of carbon electrodes.

In present work, a new way was developed for the co-production of phenol and supercapacitor electrode material from pyrolysis of biomass impregnated with NH_4_H_2_PO_4_. NH_4_H_2_PO_4_ is an inexpensive and common chemical reagent with high N and P contents. The introduction of NH_4_H_2_PO_4_ could alter the biomass pyrolysis process and also incorporate N and P in solid products. Therefore, polygeneration of phenol and supercapacitor electrode material could be achieved by the pyrolysis of NH_4_H_2_PO_4_-impregnated biomass. A lab-scale device was employed for the pyrolysis experiments. The influences of NH_4_H_2_PO_4_ to poplar (PA-to-PL) ratio and reaction temperature on the regulation of pyrolytic products were researched comprehensively. The optimal pyrolytic conditions for maximizing the phenol yield were identified. The solid products were then subjected to activation directly without additional activators to prepare NPACs.

## Materials and Methods

### Materials

Poplar without bark was sampled from Hebei province in China. The received poplar was crushed firstly and then sieved into 0.1–0.15 mm particles. The dried particles were subjected to ultimate and proximate analyses referring to the methods in the previous literature (Li et al., 2017). The concentrations of carbon, hydrogen, sulfur as well as nitrogen on dry basis were directly measured to be 49.60, 6.31, 0.10, and 0.07 wt%, respectively, while the content of oxygen (43.67 wt%) was calculated by difference. Moreover, the contents of ash (0.25 wt%), volatile (86.44 wt%), and fixed carbon (13.31 wt%) were also quantified.

NH_4_H_2_PO_4_ in analytical reagent grade was purchased from Macklin. Phenol in chromatography grade and KOH (AR, 90%) were purchased from Aladdin. Acetylene black, polytetrafluoroethylene (PTEF) and nickel foam were provided by Shanghai Saibo Chemical Co., Ltd.

The biomass samples impregnated with NH_4_H_2_PO_4_ (PBs) were prepared via the incipient wetness impregnation method. For each PB sample, the quantity of poplar and the volume of NH_4_H_2_PO_4_ aqueous solution were constant, while the concentrations of NH_4_H_2_PO_4_ in the solutions were different to ensure the mass PA-to-PL ratios of 0.2, 0.6, 1.0, 1.4, and 1.8, respectively. The mixtures were subjected successively to ultrasonic oscillation for 40 min and oven-drying at 105°C. The samples were collected in desiccators for further use. The as-obtained PBs were donated as PB0.2, PB0.6, PB1.0, PB 1.4, and PB1.8, respectively.

### Pyrolysis Experiments

**Figure 1:**
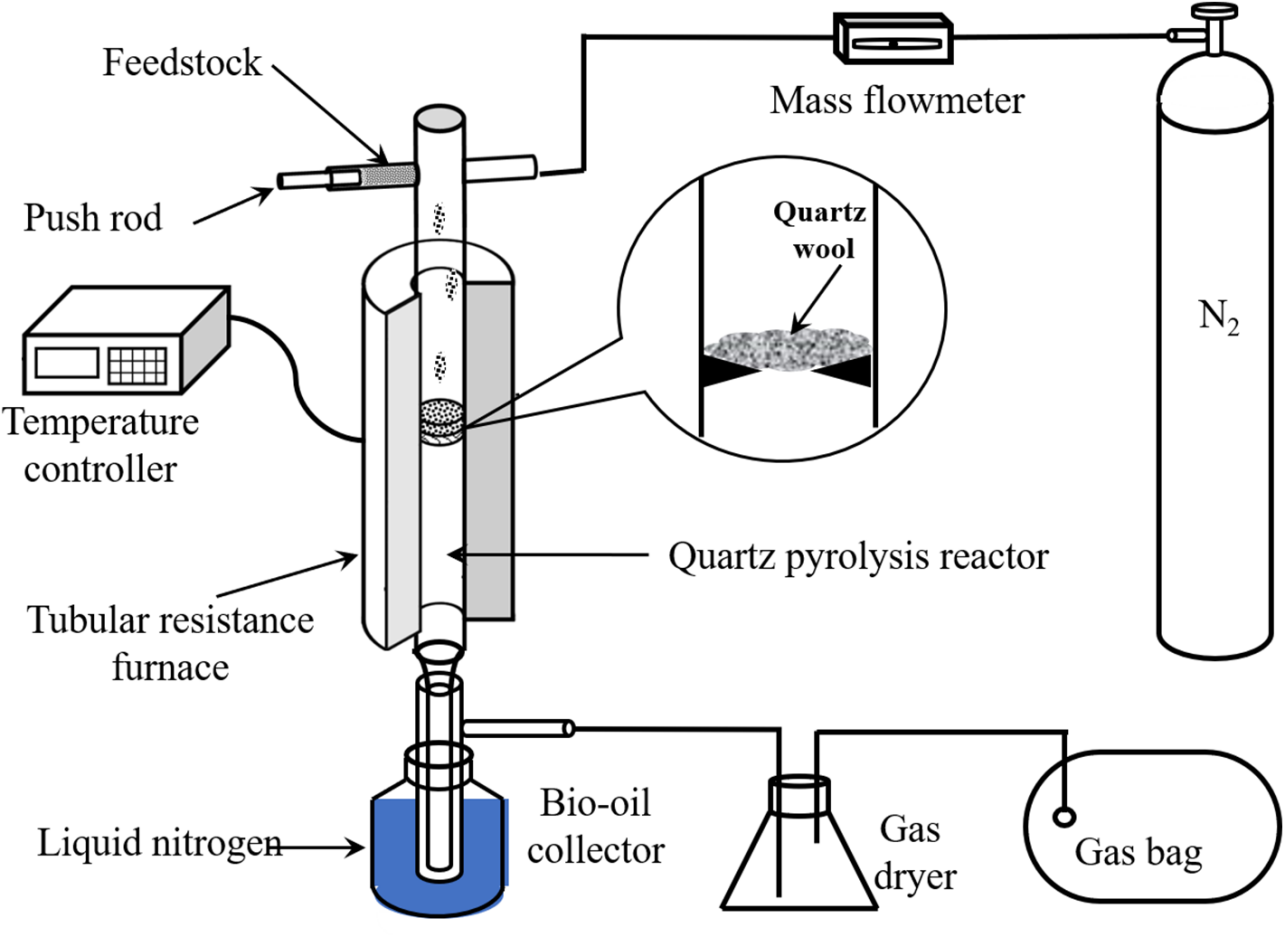
Lab-scale pyrolysis setup.

Poplar and all the PBs were pyrolyzed in a lab-scale device as illustrated in Figure 1. In brief, the device was mainly integrated with a nitrogen gas cylinder, a tubular resistance furnace, a quartz tube pyrolysis reactor, and a condenser. In each experiment, N_2_ was employed to remove air in the reactor and maintain the oxygen-free atmosphere for pyrolysis. Once the temperature of the reactor stabilized at the desired value (450, 500, 550, 600, and 650°C), the sample with a constant usage of 0.6 g was fed into the reactor continuously and uniformly in 5 min, and pyrolyzed for another 10 min.

The pyrolytic liquid and solid yields could be determined by weighing, while the gas yield was obtained by difference (Li et al., 2020). The solid product was collected from the reactor directly. Anhydrous alcohol was used to wash and dilute the liquid products. The water content was quantified using a Karl-Fisher moisture analyzer. The organic compounds were determined using a gas chromatograph instrument with their standard samples. The phenol yield was quantified by the external standard method.

### Preparation of NPAC

The solid product obtained from pyrolysis of PB0.6 at 500°C was used to prepare NPAC according to the following method. A certain amount of the solid product was firstly transferred into a tubular resistance furnace (STGX-110-12, Henan Sante Furnace Technology Co., Ltd, China). Subsequently, the solid was heated to 800°C at 5°C min^−1^, then hold for 1.5 h in nitrogen atmosphere. Following the high temperature activation, hot distilled water about 80°C was employed to wash the sample repeatedly until a neutral pH was achieved. After being subjected to drying at 110°C, the final product was obtained and donated as NPAC. In addition, the pyrolytic solid product from pyrolysis of pure poplar at 500°C was also treated with the same method, the obtained solid was used as the control group and donated as PC.

### Characterization of NPAC

An X-ray diffractometer (XRD, D8 advance) with Cu Kα radiation (λ = 0.15406 nm) was adopted to demonstrate the crystallographic structures of NPAC and PC. The data were recorded over the 2θ range of 10–90° with a scan rate of 6°/min. Chemical states of main elements (especially the N and P) on the NPAC surface were characterized by an X-ray photoelectron spectrometer (XPS, Thermo Escalab 250Xi) with Mg Kα radiation (1486.6 eV). All the binding energies were determined by referencing to the C1s peak at 284.8 eV. Porous characteristics were determined by nitrogen physisorption (ASAP2460) at 77 K. The SSA was calculated by the Barrett-Emmett-Teller (BET) method, while the pore volume and average pore diameter were determined by the Barrett-Joyner-Halenda (BJH) method. An inductively coupled plasma optical emission spectrometry (ICP-OES, Agilent ICPOES730) was adopted to measure the P content.

### Electrochemical Tests of NPAC

The electrochemical tests of NPAC and PC were conducted using a three-electrode instrument (CH Instruments 760) with 6 M KOH solution. Wherein, the platinum plate was adopted as a counter electrode, and saturated calomel as a reference electrode. The working electrode was synthetized via mixing NPAC (or PC), acetylene black and PTEF completely to homogeneous slurry at the mass ratio of 8:1:1, which was subsequently daubed on a nickel foam (1 × 1 cm) uniformly, subjected to drying at 105°C, and pressed at 10 MPa.

The cyclic voltammetry (CV) tests were executed at various scan rates of 5, 10, 20, 40, and 100 mV s^−1^. The galvanostatic charge/discharge (GCD) experiments were executed at current densities of 1, 2, 5, 10, and 20 A g^−1^. The electrochemical impedance spectroscopy (EIS) properties were tested from 10 mHz to 100 kHz in 5 mV.

## Results and Discussion

### Pyrolysis of Poplar Impregnated With NH_4_H_2_PO_4_

#### Influence of PA-to-PL Ratio on the Pyrolysis of Poplar Impregnated With NH_4_H_2_PO_4_

Poplar and PBs samples were employed for pyrolysis experiments at 500°C to elucidate the influences of PA-to-PL ratio on the distribution of main pyrolytic products (Table 1). For the pure poplar, the pyrolytic liquid yield was 50.75 wt%, accompanied with the gas and solid yields of 17.03 and 32.22 wt%, respectively. Whereas, the pyrolytic products yields changed greatly after the impregnation of NH_4_H_2_PO_4_ on the poplar. For the pyrolysis of PB0.2, the yields of liquid, gas, and solid became 36.62, 14.45, and 48.93 wt%, resulting from the combined and interacted decomposition of poplar and NH_4_H_2_PO_4_. It is notable that NH_4_H_2_PO_4_ could decompose in the pyrolytic condition to concurrently produce large quantities of water and solid products. For pure NH_4_H_2_PO_4_, its decomposition would generate around 61.74 wt% P_2_O_5_, 23.48 wt% water, and 14.78 wt% NH_3_ at the pyrolytic temperature over 210°C (Di Blasi et al., 2007), higher than the yields of solid product and water from pure poplar. Whereas, in PB pyrolysis, the NH_4_H_2_PO_4_ would not decompose singly (Li et al., 2016). Instead, it reacted with poplar decomposition products. The NH_3_ derived from NH_4_H_2_PO_4_ could react with O-containing functional groups to form N-containing compounds in the liquid product and achieve the incorporation of N in the solid product. Figure S1 shows the typical ion chromatogram of the liquid product from pyrolysis of PB0.6 at 500°C. Apparently, phenol was the dominant product. The other organic compounds were all in low contents, which were mainly N-containing heterocyclic compounds (NHCs) and furfural. The NHCs mainly included pyridine, 2-methylpyridine and 3-pyridinol, they were believed to be derived from the Maillard reaction and further condensation reactions (Li et al., 2016; Chen et al., 2018a). Moreover, the parent acid from NH_4_H_2_PO_4_ decomposition would act as the catalyst to deteriorate the dehydration and carbonization reactions in the pyrolysis process (Di Blasi et al., 2008). Considering the decomposition of NH_4_H_2_PO_4_, the yield results still indicated that the impregnation of NH_4_H_2_PO_4_ inhibited the pyrolytic devolatilization of poplar to generate liquid and gas products, while promoted the charring to obtain solid product. With the continuous increase of PA-to-PL ratio, the yields of all the products kept such trends. As a result, at PA-to-PL ratio of 1.8, the solid yield significantly rose to 67.59 wt%, accompanied with liquid and gas yields as low as 26.19 and 6.22 wt%, respectively. Moreover, the water content of liquid product increased monotonically from 34.30 wt% with pure poplar to 76.06 wt% at PA-to-PL ratio of 1.8. The increase of water content and decrease of liquid yield further confirmed the catalytic effects of NH_4_H_2_PO_4_ to enhance dehydration and charring reactions, while inhibit the generation of organic compounds (Di Blasi et al., 2008).

**Table 1:** Product distributions and phenol yields under different PA-to-PL ratios at 500°C.

**PA-to-PL ratio**	**Liquid yield (wt%)**	**Water content^a^ (wt%)**	**Gas yield (wt%)**	**Solid yield (wt%)**	**Phenol yield^b^ (wt%)**	**Phenol selectivity^c^ (%)**
0	50.75	34.30	17.03	32.22	0.49	1.47
0.2	36.62	54.94	14.45	48.93	3.07	13.67
0.6	34.10	60.34	11.86	54.04	4.57	20.09
1.0	31.87	64.61	9.61	58.52	3.04	14.23
1.4	28.31	68.98	8.28	63.41	2.51	12.13
1.8	26.19	76.06	6.22	67.59	1.48	7.61

a *Based on the pyrolytic liquid product*.

b *Based on the poplar quantity in the PBs*.

c *Percentage of phenol in the organic liquid*.

As shown in Table 1, only a little phenol was generated from pyrolysis of pure poplar with an unsatisfactory yield of 0.49 wt% and a selectivity of 1.47%, which was entirely in accordance with the previous research (Zhang et al., 2019). With the impregnation of NH_4_H_2_PO_4_, the phenol yield as well as the corresponding selectivity was increased dramatically. The phenol yield reached 3.07 wt% even at PA-to-PL ratio of 0.2. With the further rising of PA-to-PL ratio, the phenol yield exhibited a tendency of increasing first and then decreasing. At PA-to-PL ratio of 0.6, the phenol yield achieved its optimal value of 4.57 wt% with a satisfactory selectivity of 20.09%, which was 8.33 times higher than that (0.49 wt%) from pure poplar. The significant increase of phenol yield might be attributed to several reasons. Firstly, during the process of NH_4_H_2_PO_4_ impregnation, partial filaments in the poplar were cleaned, and the initial structure was changed to some extent owing to the acidity of NH_4_H_2_PO_4_ solution. Consequently, the tight linkages between lignin and other components were weakened (Di Blasi et al., 2008), resulting in easy decomposition of biomass for phenol. Besides, during the pyrolysis, NH_4_H_2_PO_4_ could act as a donor of hydrogen for biomass (Zeng and Bernstein, 2019). As confirmed in previous literatures (Lu et al., 2018c; Zhang et al., 2019), the hydrogen source would exhibit positive effects on the formation of phenol by removing the substitutes on the aromatic rings. Furthermore, the co-pyrolysis of NH_4_H_2_PO_4_ and poplar could generate abundant P-containing functional groups, like –O–P–C–, –C–P=O, and so on (Zhang et al., 2018b), which could improve the formation of phenol from non-lignin constituents, viz. cellulose. This point has been verified by previous study, wherein the AC activated by phosphoric acid was employed for phenol preparation from pyrolysis of cellulose (Zhang et al., 2018a). All of these factors might be responsible for the enhanced phenol yield. However, large PA-to-PL ratio could not improve but inhibit the formation of phenol, which might be ascribed to the exacerbated polycondensation and carbonization reactions to deteriorate the formation of all organic volatile compounds (Di Blasi et al., 2008).

#### Influence of Temperature on the Pyrolysis of Poplar Impregnated With NH_4_H_2_PO_4_

The PB0.6 sample was subjected to pyrolysis under various temperatures, i.e., 450, 500, 550, 600, and 650°C. As demonstrated in Table 2, with the temperature rising from 450 to 650°C, the liquid yield rose gradually from 31.45 to 40.40 wt%, together with the gas yield extending from 10.65 to 17.38 wt%. While the solid yield exhibited an opposite trend and decreased from 57.90 to 42.22 wt%. Meanwhile, the water content in the liquid rose from 52.53 wt% at 450°C to 69.55 wt% at 650°C. This agreed well with biomass pyrolysis characteristics under different temperatures (Zhang et al., 2012). The high pyrolysis temperature could promote the heat transfer and cracking of macromolecular materials, thereby enhancing the generation of volatile compounds and reduction of solid product.

**Table 2:** Product distributions and phenol yields at different temperatures with PA-to-PL ratio of 0.6.

**Temperature (°C)**	**Liquid yield (wt%)**	**Water content (wt%)**	**Gas yield (wt%)**	**Solid yield (wt%)**	**Phenol yield (wt%)**	**Phenol selectivity (%)**
450	31.45	52.53	10.65	57.90	4.06	16.84
500	34.10	60.34	11.86	54.04	4.57	20.09
550	36.41	65.27	14.24	49.35	3.47	16.43
600	38.31	68.75	15.75	45.94	2.83	14.72
650	40.40	69.55	17.38	42.22	2.58	11.98

With the rising of pyrolytic reaction temperature, the phenol yield exhibited a tendency of first increasing and then decreasing. A passable phenol yield of 4.06 wt% was obtained at 450°C, and further increased to an expected value of 4.57 wt% at 500°C, accompanied with an obvious increase of phenol selectivity from 16.84 to 20.09%. When the temperature was further elevated, the phenol witnessed a continuous decrease. At 650°C, the phenol yield was only 2.58 wt% with a poor selectivity of 11.98%. The results manifested that the proper low temperature could promote the generation of phenol (Chang et al., 2018), while high temperature would enhance the secondary cracking and condensation reactions induced by the acidic decomposition products of NH_4_H_2_PO_4_. Consequently, the formation of phenol was inhibited and other competitive reactions were promoted (Zhang et al., 2019).

### Physicochemical Properties of NPAC

**Figure 2:**
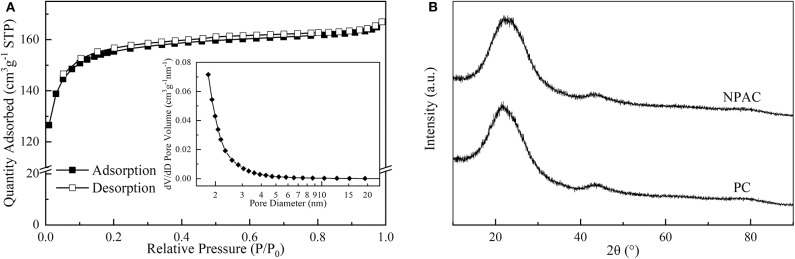
**(A)** N_2_ sorption isotherm of NPAC and **(B)** XRD patterns of NPAC and PC.

The microstructure properties of NPAC were executed, with the relevant results illustrated in Figure 2A. According to the prominent increase of N_2_ uptake capacity at low P/P_0_ values (<0.2), the N_2_ sorption isotherm plots of NPAC in Figure 2A could be classified to type I based on the IUPAC nomenclature (Thommes et al., 2015). Meanwhile, there were no obvious hysteresis loops, indicating the NPAC contained abundant micropores and a few mesopores. This could be confirmed by the pore size distribution, showing most pore sizes lower than 4 nm. In regard to the structure parameters, the SSA and total pore volume (V_total_) of PC were only 2.0 m^2^ g^−1^ and 0.008 cm^3^ g^−1^, respectively. Whereas, for the NPAC as shown in Table 3, its SSA (529.4 m^2^ g^−1^) and the V_total_ (0.258 cm^3^ g^−1^) were improved apparently, of which the micropores contributed 91.88 and 85.66%, respectively. NH_4_H_2_PO_4_ was a common activator for preparing activated carbon (Cheng et al., 2019). In this study, the pyrolysis of poplar impregnated with NH_4_H_2_PO_4_ and subsequently high temperature activation was similar to the NH_4_H_2_PO_4_ activation of poplar, resulting in the enlarged SSA and V_total_. Meanwhile, as an N and P source, the NH_4_H_2_PO_4_ could improve the N and P contents in the NPAC, about 3.75 and 3.65 wt%, respectively (Xu et al., 2017).

**Table 3:** Microstructure properties and elemental composition of NPAC.

**SSA (m**^****2****^ **g**^****−1****^**)**		**Pore volume (cm**^****3****^ **g**^****−1****^**)**		**Diameter (nm)**	**Elemental composition (wt%)**				
**S**_**BET**_	${\text{S}}_{\text{mic}}$^a^	${\text{V}}_{\text{total}}$^b^	${\text{S}}_{\text{mic}}$^c^	**D**_**average**_	**C**	**H**	**O**	**N**	**P**
529.4	486.4	0.258	0.221	1.95	66.56	1.12	19.33	3.75	3.65

a,c *t-Plot micropore area and micropore volume*.

b *P/P_0_ = 0.989*.

Figure 2B illustrates the XRD patterns of PC and NPAC. Both samples had similar patterns, there were no obvious crystal diffraction peaks but two broad peaks, a prominent one at 2θ of 23.7° and a weak one at ~43.2°. The broad peak at 23.7° was ascribed to the diffraction plane of (002), representing the graphitic stacking (Zou and Jiang, 2020). While the weak peak was ascribed to the diffraction plane of (100), indicating a limited degree of graphitization (Raj et al., 2018). The two peaks indicated that both PC and NPAC were with highly amorphous states (Wang et al., 2019).

**Figure 3:**
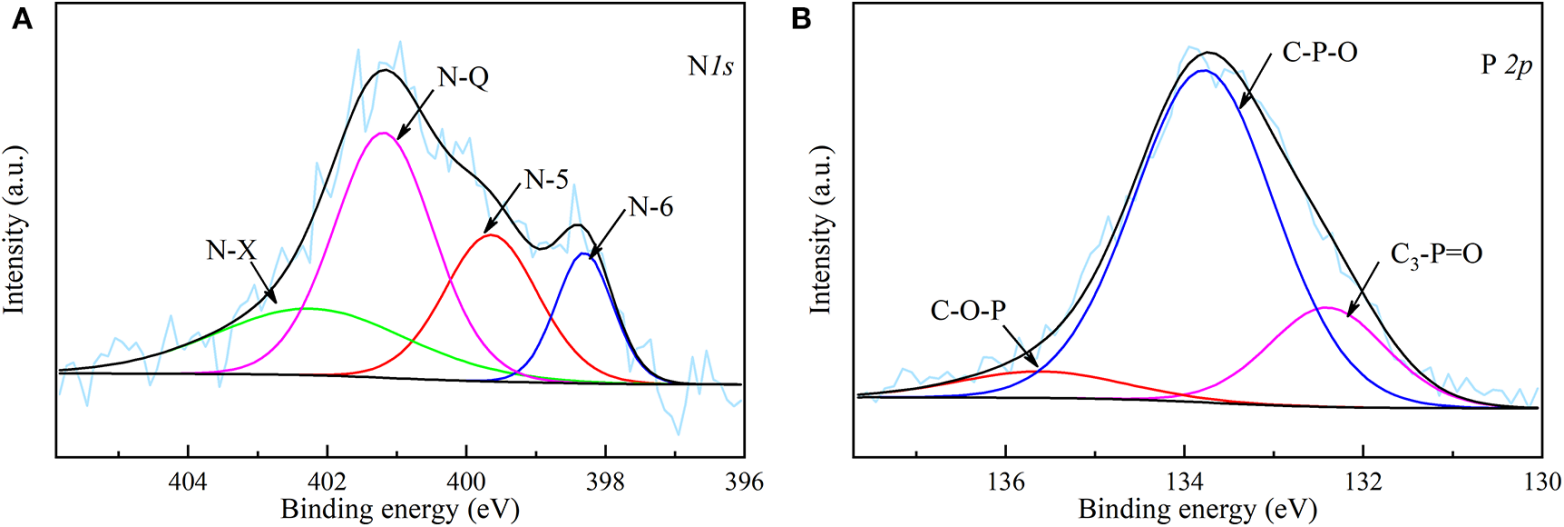
**(A)** N *1s* spectrum and **(B)** P *2p* spectrum of NPAC.

The XPS spectra of N *1s* and P *2p* of NPAC are depicted in the Figure 3. Four peaks centered at 398.30, 399.65, 401.20, and 402.39 eV were observed in the N *1s* spectrum, which represented pyridinic-N (N-6), pyrrolic-N (N-5), quaternary-N (N-Q), and oxidized N (N-X), respectively (Chen et al., 2016). As indicated above, the N in NPAC originated from the reaction of NH_3_ with O-containing groups during the pyrolysis process. The N-5 and N-6 were believed to be the cyclization products of N-containing intermediates, while the N-Q might come from the condensation reactions of N-6 (Chen et al., 2018a). These N species possessed different contributions to the electrochemical properties. In General, the N-5 and N-6 can improve the pseudocapacitance, while the N-Q can enhance the conductivity (Fu et al., 2019; Ma et al., 2020). For the P species in Figure 3B, the band at 132.4 eV was attributed to C_3_-P=O, the peaks centered at 133.78 and 135.61 eV were considered to be C–O–P and C–P–O (Liu et al., 2015). The introduction of P could not only improve the pseudocapacitance, but also widen the operating voltage window of the capacitor (Ma et al., 2018).

### Electrochemical Performance of NPAC

**Figure 4:**
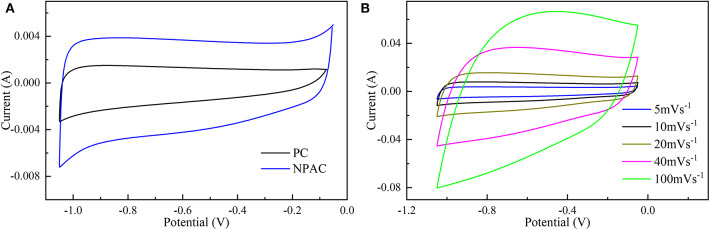
**(A)** CV curves of PC and NPAC at 5 mV s^−1^ and **(B)** CV curves of NPAC at various scan rates.

The electrochemical performances of NPAC were systemically characterized via CV, GCD, and EIS. Figure 4A illustrates the CV curves of PC and NPAC at 5 mV s^−1^. Compared with PC, NPAC possessed better rectangularity and larger surface area, resulting from its high SSA and the N and P co-doping in carbon (Wu et al., 2015). With the rise of voltage scan rates, the corresponding CV curves of NPAC still displayed similar rectangular shapes with certain deformation, as illustrated in Figure 4B. Besides, there were no obvious peaks, revealing the perfect electrochemical performance of NPAC with lower inner resistance (Li et al., 2019).

**Figure 5:**
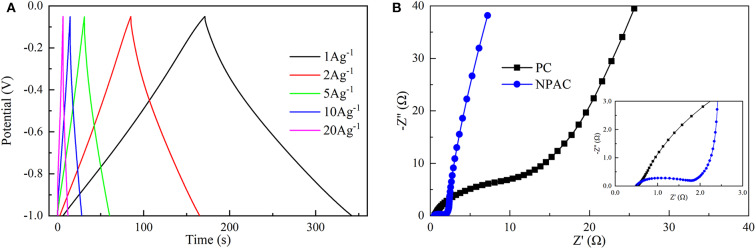
**(A)** GCD plots of NPAC and **(B)** Nyquist plots of PC and NPAC.

Figure 5A illustrates the GCD curves of NPAC obtained under various current densities (1–20 A g^−1^). Apparently, all the GCD curves exhibited high triangularity. The linear relationship between voltage and time represented excellent electrochemical capacitance behavior (Chen et al., 2018b). Further, the C_g_ values were calculated and inversely correlated with current densities. The maximum C_g_ of 181.3 F g^−1^ was obtained at 1 A g^−1^. The higher current densities (20 A g^−1^) led to an attenuation of 30% in C_g_. In other words, the capacitance retention remained about 70%, higher than many relevant values in previous researches (Wang et al., 2013a; Xu et al., 2019). This indicated the excellent electrochemical capacitive properties. Generally, under high charging/discharging current densities, the charging could finish quickly in high speed. However, the large impedance caused by the micropores in NPAC would lead to the charging time constant too long to charge completely, resulting in the decrease of C_g_s under high current densities (Divya and Rajalakshmi, 2020).

Figure 5B illustrates the Nyquist plots of PC and NPAC. Compared with PC, the NPAC possessed a semicircle with small diameter at high frequency. The diameter reflected the charge transfer resistance (R_ct_) (Wei et al., 2015), while the intersection point of the abscissa axis and semicircle represented the equivalent series resistance (ESR) (Sun et al., 2019). Apparently, the R_ct_ of NPAC was nearly 1.2 Ω, much lower than that of PC (about 22 Ω). The ESR of NPAC was about 0.5 Ω, indicating the good conductivity of NPAC (Chen et al., 2016; Zhang et al., 2017). The larger line slope of NPAC close to vertical at the low-frequency region indicated its excellent electrical capacitance characteristics (Yang et al., 2018). In brief, the high content of heteroatoms (N and P) and good porous structure enabled the NPAC with the promising potential as a supercapacitor.

## Conclusions

Pyrolysis of NH_4_H_2_PO_4_-impregnated biomass provided a promising way for polygeneration of phenol and NPAC. A lab-scale device was utilized for the experiments to reveal the effects of PA-to-PL ratio as well as pyrolytic temperature on the products formation and phenol yield. The pyrolytic solid product obtained under the maximal phenol yield condition was used to prepare NPAC by direct high temperature activation. The results indicated that the highest phenol yield attained 4.57 wt% with a satisfactory selectivity of 20.09% at 500°C and PA-to-PL ratio of 0.6. The as-obtained NPAC exhibited promising electrochemical performance, with an excellent C_g_ of 181.3 F g^−1^ at 1 A g^−1^, and thus, could be used as a supercapacitor electrode material.

## Data Availability Statement

The raw data supporting the conclusions of this article will be made available by the authors, without undue reservation, to any qualified researcher.

## Author Contributions

KL: conceptualization, methodology, and writing-original draft. BW: investigation, and writing-review and editing. DB: methodology. DN: investigation and validation. QL: conceptualization, supervision, writing-review and editing, and funding acquisition.

## Conflict of Interest

The authors declare that the research was conducted in the absence of any commercial or financial relationships that could be construed as a potential conflict of interest.
