# Supplementary material for: Pyrolysis of Biomass Impregnated With Ammonium Dihydrogen Phosphate for Polygeneration of Phenol and Supercapacitor Electrode Material
Source: Front Chem. 2020 May 19;8:436. doi: 10.3389/fchem.2020.00436 (PMC7248177; doi:10.3389/fchem.2020.00436)
Supplement: Supplementary file 1 [file Table_1.DOCX]

Supplementary Material

**GC/MS analysis of the liquid product**

The main organic compounds in the liquid product were analyzed in a PerkinElmer GC/MS (Clarus 560) with helium (99.999%) as the carrier gas. The volatile products were separated on a TG-5MS capillary column (30 m× 0.25 mm ×0.25 μm) and identified according to the NIST library. The GC oven rose from 40°C to 280°C at 15°C/min, and then maintained for 2 min. The temperatures of the transmission line and GC inlet were set as 300°C, and the temperatures of GC/MS interface and ion source were stabilized at 280°C.





**Figure S1.** Typical ion chromatogram of the liquid product from pyrolysis of PB0.6 at 500°C

1: pyridine; 2: 2-methylpyridine; 3: furfural; 4: phenol; 5: 3-methylphenol; 6: 3-pyridinol; 7:2,6-dimethoxyphenol
